# How Metabolic Rate Relates to Cell Size

**DOI:** 10.3390/biology11081106

**Published:** 2022-07-25

**Authors:** Douglas S. Glazier

**Affiliations:** Department of Biology, Juniata College, Huntingdon, PA 16652, USA; glazier@juniata.edu

**Keywords:** allometric scaling, body size, cell expansion and multiplication, cell size, geometry and composition, hierarchical effects, rates of growth and metabolism

## Abstract

**Simple Summary:**

The metabolic conversion of resources into living structures and processes is fundamental to all living systems. The rate of metabolism (‘fire of life’) is critical for supporting the rates of various biological processes (‘pace of life’), but why it varies considerably within and among species is little understood. Much of this variation is related to body size, but such ‘metabolic scaling’ relationships also vary extensively. Numerous explanations have been offered, but no consensus has yet been reached. Here, I critically review explanations concerning how cell size and number and their establishment by cell expansion and multiplication may affect metabolic rate and its scaling with body mass. Numerous lines of evidence suggest that cell size and growth can affect metabolic rate at any given body mass, as well as how it changes with increasing body mass during growth or evolution. Mechanisms causing negative associations between cell size and metabolic rate may involve reduced resource supply and/or demand in larger cells, but more research is needed. A cell-size perspective not only helps to explain some (but not all) variation in metabolic rate and its body-mass scaling, but may also foster the conceptual integration of studies of ontogenetic development and body-mass scaling.

**Abstract:**

Metabolic rate and its covariation with body mass vary substantially within and among species in little understood ways. Here, I critically review explanations (and supporting data) concerning how cell size and number and their establishment by cell expansion and multiplication may affect metabolic rate and its scaling with body mass. Cell size and growth may affect size-specific metabolic rate, as well as the vertical elevation (metabolic level) and slope (exponent) of metabolic scaling relationships. Mechanistic causes of negative correlations between cell size and metabolic rate may involve reduced resource supply and/or demand in larger cells, related to decreased surface area per volume, larger intracellular resource-transport distances, lower metabolic costs of ionic regulation, slower cell multiplication and somatic growth, and larger intracellular deposits of metabolically inert materials in some tissues. A cell-size perspective helps to explain some (but not all) variation in metabolic rate and its body-mass scaling and thus should be included in any multi-mechanistic theory attempting to explain the full diversity of metabolic scaling. A cell-size approach may also help conceptually integrate studies of the biological regulation of cellular growth and metabolism with those concerning major transitions in ontogenetic development and associated shifts in metabolic scaling.

## 1. Introduction

Metabolism constitutes the collective biochemical processes by which organisms transform environmental resources into various biological structures and processes. Accordingly, the rate of metabolism relates to the pace of many kinds of biological processes, and thus its variation may have multiple effects on the temporal dynamics of the physiology, development, behavior, evolution, and ecological interactions of organisms [1,2,3,4]. Therefore, factors causing variation in metabolic rate have long interested many kinds of biologists. A major intrinsic factor related to metabolic rate (*R*) is body mass (*M*), which has often been described by a simple power function, as follows:

*R* = *aM^b^*, (1)

where *a* is the scaling coefficient (or antilog of the intercept in a log-log plot) and *b* is the scaling exponent (or loglinear slope). In many species and higher taxa, most of the variation in metabolic rate relates to body mass, but many other intrinsic and extrinsic factors can have significant effects as well (e.g., [2,5,6,7]), for reasons that have been debated for decades [6,7,8,9,10,11,12,13,14,15,16,17,18,19,20]. Many kinds of theories and hypotheses have been proposed, but no consensus has yet been reached, especially with respect to the causes of the body-mass scaling of metabolic rate (see e.g., [8,9,10,13,14,15,17,18,20]). Of these causes, growing interest has been shown regarding how various components of body size in multicellular organisms, such as cell size [9,14,18,20,21,22,23,24,25,26,27,28,29,30,31,32,33,34,35,36,37,38,39,40,41,42] and organ size [9,14,17,18,20,34,43,44,45,46,47,48,49], may influence variation in metabolic rate and its scaling with body mass. Since cells are where metabolism happens, it seems natural to explore how the properties of cells may affect metabolism at the tissue, organ, and whole-body levels. Cells are not only the ‘building blocks of life’, but also the ‘energy factories of life’. Hence, the purpose of my essay is to review critically the many ways (some novel) that variation in whole-body metabolic rate may relate to cell size. By doing so, I hope to stimulate further research that will explicate the mechanisms involved.

## 2. Major Ways That Variation in Metabolic Rate May Relate to Cell Size

Variation in metabolic rate may relate to cell size in multiple ways, which are summarized in Table 1. Each of these ways is briefly described in the following sections, including pertinent theory and data, as available.

### 2.1. Mass-Specific or Mass-Corrected Metabolic Rate

#### 2.1.1. Theory

Simple geometric surface area (SA) theory predicts that organisms with relatively large cells should have lower mass-specific metabolic rates (i.e., lower metabolic rates per unit mass, *R*/*M*, derived by simple ratio calculations or other types of mass correction that account for allometric relationships between *R* and *M*; for a recent review, see [7]) than organisms with relatively small cells [20,22,23,25,26,27,28]. This hypothesis assumes that metabolically important processes, such as resource uptake and metabolic waste removal, are more limited by the smaller amounts of available cell SA relative to cell or tissue volume (V) in organisms with large vs. small cells, assuming no significant differences in cell shape (i.e., cell isomorphy) [14,22,23,53]. In addition, the energetic costs of maintaining ionic gradients across cell membranes may be smaller per unit cell area in relatively large cells [20,23,25,27]. Other possible mechanisms related to cell size (i.e., intracellular resource transport, and cell composition and multiplication) are considered in Section 2.3.2, Section 2.3.3, Section 3.2, Section 3.3, and Section 3.4.

#### 2.1.2. Interspecific Patterns

As expected from predictions of cell SA theory, multiple studies have reported negative associations between *R*/*M* and cell size (or its proxy, genome size) among related animal species, including carabid beetles [41], amphibians [22,28,54], eyelid geckos [32], birds [28,40,55], and mammals [26] (for recent reviews, see [20,56,57]). Most of these studies have focused on the sizes of erythrocytes (red blood cells), which are importantly involved in oxygen exchange in respiratory systems and thus may be linked to *R*/*M* for this reason. However, associations between *R*/*M* and other types of non-respiratory cells (e.g., in ommatidia and Malphigian tubules of carabid beetles [41]) have also been found, and thus cell size may have a general effect on organismal metabolic rate, which requires further research (see also Section 2.1.3, Section 2.2, Section 2.3, and Section 3).

#### 2.1.3. Intraspecific Patterns

Like interspecific comparisons, intraspecific comparisons of *R*/*M* with cell size often show significant negative relationships [33,39,55,58,59,60,61,62,63], but contrary to cell SA theory, nonsignificant [39,61,64,65,66,67] and even positive [42,56,59,60,63,68] relationships have also been frequently reported. Why intraspecific relationships vary so much is little understood, but differences in temperature [28,54,59,61,63], fasting duration [68], developmental stage [39], tissue type [40,60], and duration of laboratory acclimation [61,63] or evolutionary adaptation [54,56,69] may be at least partially involved.

Since cell size for a given tissue type tends to vary less within than among species, predicted cell-size effects may be more difficult to detect within species because they are more easily obscured by the effects of other extraneous factors. However, intraspecific studies of temperature effects largely support cell SA theory, because increasing temperature tends to cause both an increase in metabolic rate and a decrease in cell size (thus increasing cellular SA/V ratios that can better accommodate an increasing metabolic demand) (reviewed in [56]; but see [70]), as predicted.

### 2.2. ‘Metabolic Level’ or Vertical Elevation of a Body-Mass Scaling Relationship for Metabolic Rate

**Figure 1:**
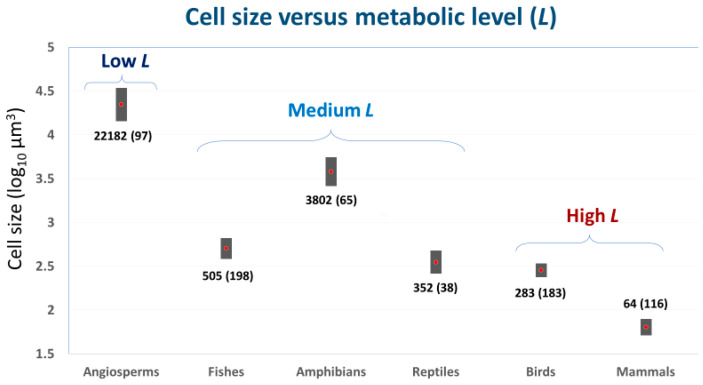
Means (red dots) and ranges (dark vertical bands) of cell size (log_10_ µm^2^; data from [71]) in relation to metabolic level (*L*) of six major taxonomic groups of multicellular organisms. Mean cell sizes (number of species sampled in parentheses) are indicated (based on epidermal cells in the angiosperms, and erythrocytes in the animal taxa). Low *L* ≈ −2.3; medium *L* ≈ −1.5 to −1.1; high *L* ≈ 0 to −0.5 (*L* = log_10_ mL O_2_ g^−1^ h^−1^ at midpoint of log_10_ body-mass range; data from Figure 3 in [29]). Note the apparent negative correlation between cell size and *L*, as expected from cell-size surface area theory (see text). The especially small cell sizes observed for mammals may be in part due to their erythrocytes having no nuclei. Cell size also appears to be positively related to the metabolic scaling exponents (*b*) of these taxa (angiosperms: 1.06; fishes: 0.88; amphibians: 0.88; reptiles: 0.76; birds: 0.64; mammals: 0.68; data from [29,50]; see also Section 2.3.5).

Cell size may also relate to the overall metabolic rate of conspecific or heterospecific animals with different body masses (=metabolic level, *L*, as estimated by *R*/*M* at the geometric midpoint of a scaling relationship between log *R* and log *M*; see e.g., [29,50,51,52]). For example, species-specific *L* values of non-polyploid teleost fishes are significantly negatively related to red blood cell size [29], as expected by cell SA theory. Similarly, among major taxa of eukaryotic organisms, *L* appears to be negatively related to mean cell size (Figure 1). Low-*L* angiosperm plants have cells with average volumes ~80 to 350 times larger than those of high-*L* endothermic birds and mammals, whereas medium-*L* ectothermic vertebrates have cells with intermediate volumes. Further research is needed to test the strength and generality of these relationships.

### 2.3. Slope of a Body-Mass Scaling Relationship for Metabolic Rate

#### 2.3.1. Effects of Cellular Mode of Growth (Cell Expansion versus Multiplication)

**Figure 2:**
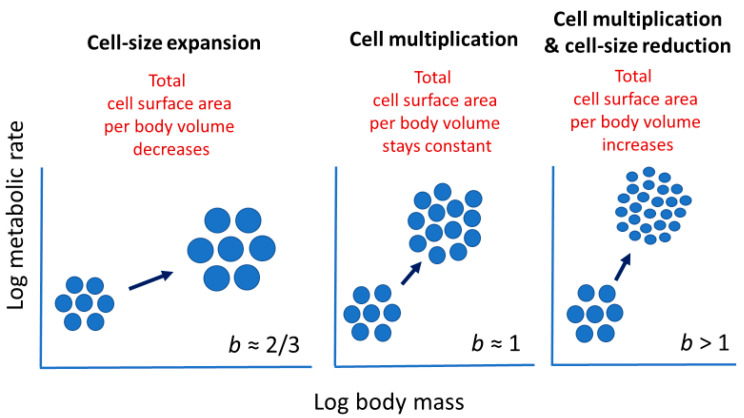
Schematic representation of how various cellular modes of body growth (cell-size expansion, cell multiplication, and cell multiplication with cell-size reduction) should affect the ontogenetic scaling of metabolic rate with body volume or mass (scaling exponent *b* = loglinear slope), according to cell-size metabolic scaling theory (see text and [23,24,27]). If both cell expansion and multiplication occur during growth, *b* should be between 2/3 and 1.

Cell size may relate to not only the vertical elevation of a metabolic scaling relationship (*L*, see Section 2.2), but also its slope (i.e., scaling exponent *b* in Equation (1)). Cell-size metabolic scaling theory [23,24,27] predicts that if organismal growth occurs entirely through cell expansion (hypertrophy), *b* should be 2/3 (because total cell surface area supporting metabolic activity should scale with body volume or mass to the 2/3 power). By contrast, if growth occurs entirely through cell multiplication with no change in cell size (hyperplasia and isotrophy), *b* should be 1, because total cell surface area should scale isometrically with body volume or mass. Therefore, growth by both cell expansion and multiplication should result in *b* values between 2/3 and 1 (Figure 2). Another possible option not recognized previously by cell-size metabolic scaling theory is cell multiplication coupled with cell-size reduction (hyperplasia and hypotrophy, resulting from cell division with little gain in total biomass), which should cause *b* > 1 (Figure 2). For all possible options, one can calculate the predicted metabolic scaling slope *b* by using the equation

*b* = 1 − *c*/3, (2)

where *c* equals the slope for log cell size (area, volume, or mass) in relation to log total tissue, organ, or body size (area, volume, or mass) [72].

Tests of cell-size metabolic scaling theory have involved comparisons of both intra- and interspecific metabolic scaling relationships. Davison pioneered this kind of approach by showing that the *b* values of the *R-M* scaling relationship match those expected from the *M* scaling of muscle cell size in the frog *Rana pipiens* [23] and of ommatidial and muscle cells in the crayfish *Procambarus alleni* [24]. As a result, the scaling of metabolic rate paralleled the scaling of total cell surface area in muscle or eye tissues. Scaling exponents in various ant species also appear to vary as expected from the intraspecific scaling of cell size (using eye-facet size as a proxy for cell size) [31]. Other studies have provided mostly positive [9,14,29,32,33,35,36,42,73,74] but sometimes negative [34,75,76] support for effects of cell size on intraspecific ontogenetic metabolic scaling.

**Figure 3:**
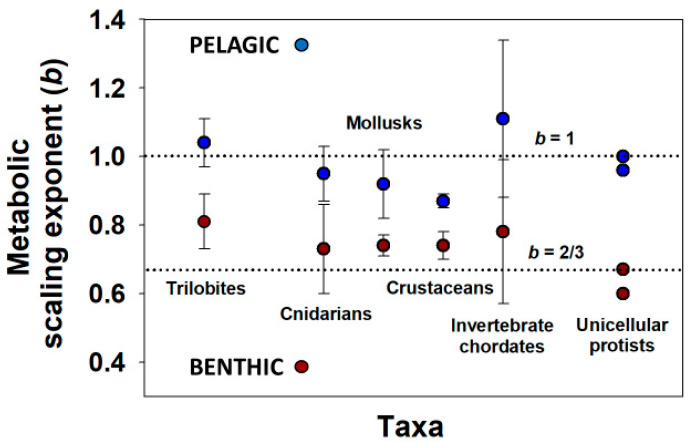
Means (±95% confidence intervals) of intraspecific metabolic scaling exponents (*b*) for various taxa of pelagic and benthic animals and protists. The trilobite *b* values were estimated using the eye-facet method of [24,31] and cell-size metabolic theory [23,24,27] (data from [72]). The *b* values for various taxa of invertebrates [80] and for the pelagic protist *Didinium nasutum* [88] and the benthic protist *Stentor coeruleus* [89] at two different temperatures are based on actual measurements of oxygen consumption rate, a proxy for metabolic rate. Note that the *b* values for pelagic species tend to be near 1, whereas those for the benthic species tend to be closer to 2/3. Some of these differences may relate to extensive use of cell multiplication during growth of pelagic species versus extensive use of cell expansion during growth of benthic species, a hypothesis that requires testing (see also text).

Cell-size metabolic scaling theory has even been used to predict *b* values for extinct pelagic (open-water) and benthic (bottom-dwelling) trilobites (again by using eye-facet size as a proxy for cell size [72], following [24,31]) that parallel those of living aquatic invertebrates and protists with pelagic (*b* ≈ 1) versus benthic lifestyles (*b* < 1) (Figure 3). It would be instructive to determine whether cell multiplication generally dominates the ontogenetic growth of pelagic species (whereas cell expansion is more important in benthic species), as appears to have occurred in trilobites [72]. In support, pelagic squid and jellyfish with *b* values often near 1 tend to grow throughout life largely by cell multiplication [9,77,78,79,80], whereas nematode worms with *b* values near 2/3 grow chiefly by cell expansion [9,81,82]. In addition, as predicted by cell SA metabolic theory, benthic ascidians (tunicates) with *b* values between 2/3 and 1 (mean = 0.78 [80]) exhibit both cell multiplication and enlargement during growth [83]. More studies are now needed to determine whether different patterns of the cellular mode of growth are generally related to differences in the metabolic scaling between pelagic and benthic species. However, the observation that even unicellular protists show pelagic versus benthic differences in metabolic scaling (Figure 3) suggests that cellular mode of growth is not the only mechanistic factor underlying these ecological effects. In addition, contrary to cell-size metabolic scaling theory, in aquatic crustaceans, eye-facet size (a proxy for cell size) scales strongly with total eye size (a proxy for body size) in both pelagic species with isometric or near-isometric ontogenetic metabolic scaling (*b*~1) (e.g., *Daphnia* [84,85,86]) and largely benthic species with allometric metabolic scaling (*b* < 1) (e.g., *Gammarus* [75]). Perhaps natural selection for visual acuity or light sensitivity may obscure relationships between eye-facet size (and thus ommatidial cell size) and metabolic scaling (see e.g., [72,87]). Further work should examine other kinds of cells. Note, however, that parallel scaling of cell size in ommatidia and other tissues has been found in the crayfish *Procambarus alleni* (muscle tissue [24]) and carabid beetles (Malphigian tubules [41]).

Inverse correlations between the interspecific *M* scaling of basal metabolic rate and genome size (a proxy for cell size) among various orders of birds and mammals also provide support for cell-size metabolic scaling theory [27]. Further evidence includes a close match between the predicted and observed *b* values for the interspecific metabolic scaling relationship of insects, based on using the eye-facet size method [72]. In addition, the hypometric scaling of metabolic rate (*b* < 1) among carabid beetles species appears to be related to larger species having larger cells in their ommatidia and Malphigian tubules than do smaller species [41]. Finally, although not previously recognized, the frequent observation of hypermetric scaling of metabolic rate (*b* > 1) in embryos and initial postembryonic stages of various kinds of animals and plants [9,14,90,91,92,93,94,95,96,97,98,99] is consistent with cell-size metabolic scaling theory, because embryonic and initial postembryonic development often involves prolific cell multiplication with relatively little or no gain in biomass [100,101], thus generating smaller cells with greater total SA per embryo V that can support a higher *R*/*M* (see Figure 2).

#### 2.3.2. Effects of Cellular Mode of Growth (Ontogenetic Shifts in Growth Rate and Metabolic Scaling)

**Figure 4:**
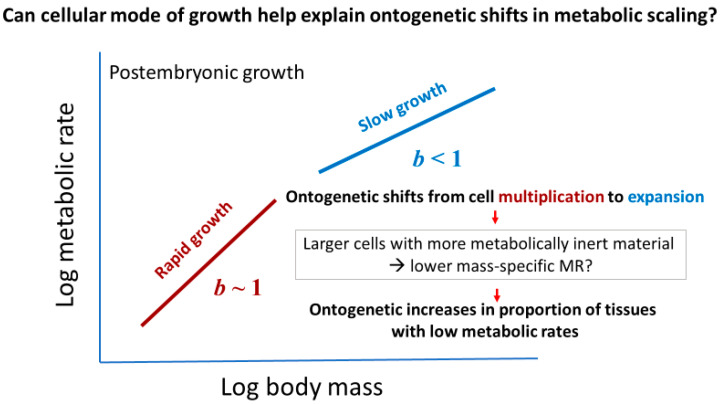
Ontogenetic shifts in metabolic scaling tend to be associated with rapid growth in early postembryonic development and slower growth in later development [9,14]. Additional possible processes involved in these ontogenetic shifts are changes from mainly cell multiplication to cell expansion and increases in proportions of tissues with relatively low metabolic rates [9,14]. These two processes may be linked, at least in part, by the ontogenetic development of larger cells with more metabolically inert materials, as in animal adipose tissues and structural tissues in vascular plants (see also Figure 6).

Many kinds of organisms show ontogenetic shifts in metabolic scaling (e.g., [9,72,91,93,95,97,98,99,102,103,104,105,106,107]). Most of these shifts involve relatively steep metabolic scaling during early postembryonic development and shallower scaling during later development (i.e., the Type III metabolic scaling described in [9]). These shifts are commonly attributed to changes in growth rate, a metabolically expensive process [3,9,14,98,102,103,105,108,109,110]. Rapid growth during early postembryonic development should result in steeper increases in metabolic rate than does slower growth during late development (Figure 4). In general, steeper ontogenetic metabolic scaling does tend to be associated with more rapid growth rates (see [75,81,98,102,103,105,108,109,110] and several other studies cited in [3,9,14,18]). Cell-size effects may also be involved, because organisms with small cells tend to grow faster than those with large cells [20], and more rapid growth tends to involve increases in cell multiplication relative to cell expansion ([111,112,113,114,115,116,117,118,119], but see [120]). Somatic growth is enhanced more by cell multiplication than cell expansion, because the former increases the amount of DNA needed for informing biosynthesis, whereas the latter does not, unless accompanied by intracellular DNA replication without cell division (i.e., endoreplication) [121].

Accordingly, early rapid growth should be associated with cell multiplication or a combination of both cell multiplication and expansion, whereas later slower growth should be associated mainly with cell expansion, as is seen in angiosperms, trilobites, tunicates, fishes, and mammals for many kinds of non-regenerating organs [9,72,113,122,123,124,125,126,127,128,129,130,131,132,133].

Consequently, enhanced nutrition in laboratory rats increases early growth by cell multiplication but later growth by cell expansion [125,134,135]. In addition, it has been proposed that selection for rapid early growth in mammals occurs primarily by increases in cell multiplication, whereas selection for increases in later growth occurs chiefly by cell expansion [136,137,138]. However, the generality of such intriguing patterns remains to be determined. Apparent exceptions include fruit flies (*Drosophila*), where rapid larval growth occurs mainly by cell expansion [81,120], and many cephalopods that grow rapidly throughout life, largely by cell multiplication [77,78] (see also Section 2.3.1).

**Figure 5:**
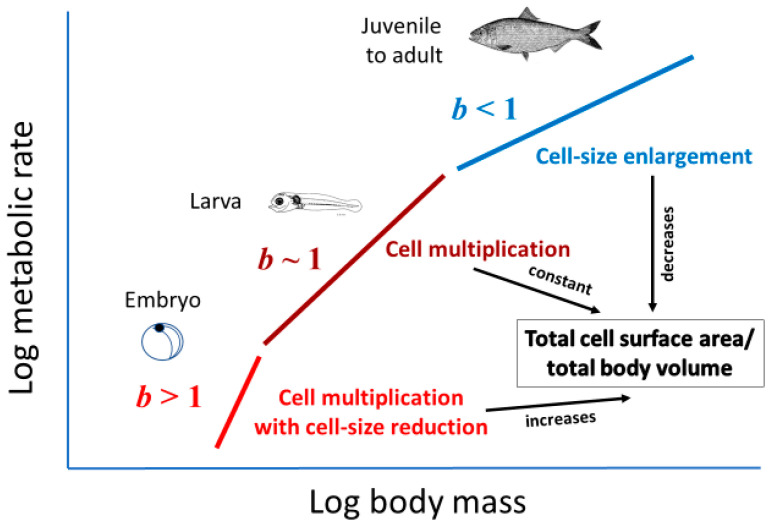
Ontogenetic shifts in metabolic scaling from egg to adult may relate to changes in the cellular mode of growth. During embryonic development, cell multiplication proceeds rapidly with little or no gain in biomass, thus causing large increases in total cellular SA relative to total biomass, thus enabling increases in mass-specific metabolic rate that result in hypermetric metabolic scaling (metabolic scaling exponent *b* > 1: red line). During early postembryonic development, rapid cell multiplication continues, but with parallel rapid gains in biomass, which enables total cellular SA relative to total biomass to stay approximately constant, thus supporting isometric metabolic scaling (*b*~1, brown line). During late postembryonic development, cell multiplication ceases or diminishes greatly, whereas cell expansion dominates somatic growth, such that total cellular SA relative to total biomass decreases, thus resulting in hypometric metabolic scaling (*b* < 1: blue line). See also Figure 2. Fish larva drawing from [139]. Adult fish drawing from [140].

The cellular mode of growth may not only explain shifts in metabolic scaling from near-isometric (*b*~1) to hypometric (*b* < 1) during postembryonic development, but also hypermetric metabolic scaling (*b* > 1) often observed during embryonic and/or very early postembryonic development (see Section 2.3.1). During the earliest stages of ontogeny, cell multiplication typically occurs with little or no gain in biomass, thus creating many small cells with high SA/V ratios that can sustain high *R*/*M*. Therefore, the cellular mode of growth may help to explain variation in metabolic scaling throughout ontogeny from egg to adult (Figure 5).

#### 2.3.3. Effects of Cellular Mode of Growth (Ontogenetic Development of Larger Cells with Large Amounts of Metabolically Inert Materials)

Since the 1930s, a major explanation of the hypometry of intra- and interspecific metabolic scaling (*b* < 1) has been that increased body size involves a disproportionate increase in the size of organs and tissues with low metabolic rates relative to those with high metabolic rates [9,14,17,18,20,34,43,44,45,46,47,48,49,76,141,142,143,144]. For example, during fish development, the mass of low-energy tissues, such as those composing the musculoskeletal system, increases relative to that of high-energy organs, such as the brain, heart, and gastrointestinal tract [34,44,45,76,144].

Here, I hypothesize that some of the ontogenetic increase in the relative masses of tissues with relatively low metabolic rates may be linked to increases in cell size, as observed during late growth stages of fat, skeletal muscle, and/or structural tissues that have lower energetic costs of maintenance than many other kinds of tissues that are routinely more active metabolically. Consider that skeletal muscle tissue, which has low metabolic costs during resting, makes up a large proportion of total body mass in many kinds of animals (~33–68% in fishes [145], and ~21–61% in mammals [145,146]), and during ontogeny, muscle growth usually initially results mainly from cell multiplication but later chiefly from cell enlargement [81,113,147,148,149,150,151], though animals with indeterminate (post-maturational) growth, such as many fishes, may show substantial cell multiplication throughout life [113,152,153]. Therefore, cell expansion via protein accumulation may be importantly involved in ontogenetic increases in relative muscle mass, and this effect may be enhanced by increased locomotor activity (exercise) [154]. In some muscle types, increased cell size may also be associated with the accumulation of metabolically inert glycogen and lipid deposits [155,156,157], but the generality of this pattern requires further substantiation.

**Figure 6:**
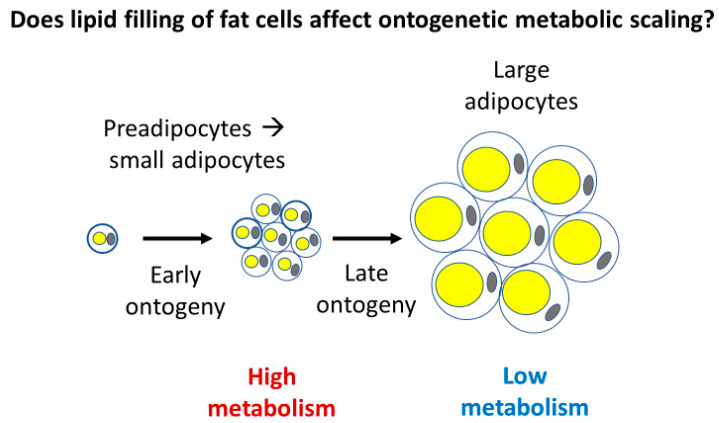
Schematic representation of the two-phase ontogeny of adipocytes (fat cells). Early in ontogeny, many small fat cells are produced, which according to cell surface area theory should have relatively high mass-specific metabolic rates (see Section 2.1.1). Later in ontogeny, these small fat cells enlarge as they become filled with lipid deposits, which should have a relatively low mass-specific metabolic rate due to their reduced surface area per volume and the accumulation of metabolically inert fat deposits (see Section 2.3.3). Hypothetically, the development of many large adipocytes with metabolically inert fat deposits should help cause a lower whole-body mass-specific metabolic rate, and thus contribute to a hypometric ontogenetic body-mass scaling of metabolic rate (slope *b* < 1).

Adipose tissue may also make up a substantial proportion of total body mass in animals (~5–45% in mammals [145,148,158]), and like muscle, its ontogenetic development first largely entails cell multiplication, but later largely cell expansion, as observed in chickens [159] and laboratory rats [160] and mice [161]. The second phase of adipose tissue development involves increased lipid deposition in fat cells (Figure 6) [159,160,161].

Similarly, in woody plants, the ontogeny of structural wood tissue, which makes up a large amount of total biomass, especially in mature individuals (e.g., large trees), involves a two-phase process of cell multiplication followed by cell expansion [162]. Furthermore, cell enlargement in woody plants involves increased lignification and/or the formation of relatively large water-filled vacuoles that provide needed turgor pressure, both of which increase structural support.

Consequently, accumulation of tissues with relatively low metabolic rates during ontogeny may importantly involve cell expansion. This may happen in two ways. First, during late ontogeny, growth in the mass of low-energy fat, muscle, and structural tissues may occur chiefly via cell expansion. Second, cell expansion in animal adipose tissues and woody plant structural tissues further entails the accumulation of metabolically inert materials (e.g., fat, lignin, and water). As a result, cell expansion may contribute to the hypometry of ontogenetic metabolic scaling (*b* < 1) by being associated with decreased *R*/*M* in at least three ways: (1) reduced cellular SA/V ratios (see Section 2.1.1 and Section 2.3.1), (2) slower somatic growth (see Section 2.3.2), and (3) the accumulation of metabolically inert materials (see also Figure 4).

#### 2.3.4. Effects of Total Cellular Surface Area in Body (at Constant Metabolic Level)

Another proposed mechanism by which cell size may affect ontogenetic metabolic scaling is via effects of total cellular surface area in the body (see also Section 2.3.1 and Section 2.3.2, and Figure 5). According to this mechanism, when *L* is constant, species with small cells, and thus relatively high total cellular SA relative to total body V or *M*, should exhibit higher metabolic scaling exponents (*b*) than species with large cells, and thus relatively low total cellular SA relative to body V or *M* [36]. This hypothesis assumes that SA effects on metabolic scaling will be stronger in species with large cells (with *b* approaching 2/3), whereas V effects will be stronger in species with small cells (with *b* approaching 1). This hypothesis is supported by a significant negative relationship between *b* and cell size observed among cyprinid fish species with similar *L* [36]. However, this hypothesis assumes that cell size does not affect *R*/*M* or *L*, which it often does (see also Section 2.1, Section 2.2, and Section 2.3.6). Therefore, I consider possible interactive effects of cell size and *L* on *b* in the next section.

#### 2.3.5. Effects of Both Cellular and Whole-Body Surface Areas (Mediated by Variable Metabolic Level)

By combining negative effects of cell size on metabolic level (*L*; see Section 2.2) and of *L* on the scaling exponent (*b*) for resting metabolic rate (following the metabolic-level boundaries hypothesis, MLBH, which has received extensive support; see e.g., [9,18,29,50,51,163,164,165,166,167,168]), Glazier [29,72] postulated that *b* should be positively correlated with cell size among species with different *L* values, which was verified in an analysis of 22 species of non-polyploid teleost fishes (*r* = 0.520; *p* = 0.013 [29]). This hypothesis assumes that lower *L* is associated with larger cells having reduced SA/V ratios that limit metabolic rate (see Section 2.2). Furthermore, as *L* decreases, whole-body V effects on metabolic rate should increase relative to SA effects, and thus *b* should increase from a minimal value of 2/3 to a maximal value of 1 in isomorphic organisms. Therefore, increased cell size should be associated with larger *b* values, as observed in fishes, and a comparison of the six major taxa of animals and plants depicted in Figure 1 (*r* = 0.897; *p* = 0.039; for *b* vs. log cell area). In short, this hypothesis shows how the combined effects of SA at the cellular and whole-body levels may affect the body-mass scaling of metabolic rate. However, note that this positive correlation is expected only if *L* varies substantially among species. No correlation or even a negative correlation may arise if *L* shows little or no variation among species, as also observed in fishes [36] (see also Section 2.3.4). In fact, controlling for variation in *L* by using a partial correlation analysis, based on the data for 22 fish species presented in [29], reveals that the positive correlation between *b* and cell size is weaker (*r* = 0.336) and no longer significant (*p* = 0.138). According to [36], when *L* is constant or controlled, *b* may become more influenced by SA effects at the cellular level relative to those at the whole organism level, a hypothesis that requires testing (see also Section 2.3.6).

#### 2.3.6. Summary of Potential Effects of Cell Size on Metabolic Scaling and Their Logical Consistency

**Figure 7:**
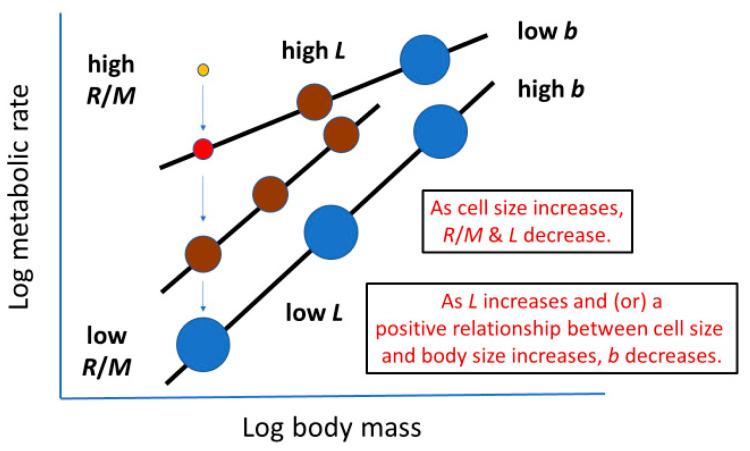
Schematic representation of how cell size (depicted by sizes of circles) may affect *R*/*M* (metabolic rate of an individual organism or species at a specific body mass), *L* (metabolic level or log mass-specific metabolic rate at the midpoint of the log body-mass range; as such, this measure represents the vertical elevation of a metabolic scaling relationship) and *b* (the loglinear slope of the scaling relationship). Note that all of the effects depicted are logically consistent and thus compatible with each other.

As can be seen, cell size can affect metabolic rate and its scaling with body mass in multiple ways (as summarized in Table 1 and Figure 7). Cell size may affect the *R*/*M* of individual species, as well as the slope (*b*) and vertical elevation (i.e., metabolic level, *L*) of intra- or interspecific metabolic scaling relationships. As shown in Figure 7, these various effects are logically consistent. Within this conceptual framework, increases in cell size are always associated with decreases in metabolic rate.

**Figure 8:**
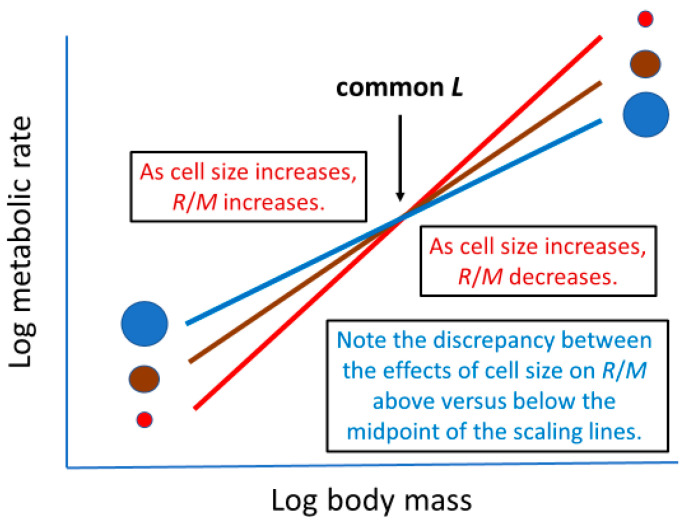
Schematic representation of how cell size (depicted by sizes of circles) may affect the metabolic scaling slope (*b*) when comparing scaling relationships with the same *L* (metabolic level or log mass-specific metabolic rate at the midpoint of the log body-mass range) and thus common midpoint (according to the mechanism posited by [36]). Note the discrepancy between the effects of cell size on *R*/*M* (mass-specific metabolic rate) above versus below the midpoint of the scaling lines.

However, the cell-SA mechanism proposed by [36], which posits a negative effect of cell size on *b* when *L* is constant, is not entirely consistent with the various effects depicted in Figure 7. Consider three hypothetical metabolic scaling relationships with identical *L* values, but different slopes (*b*) (Figure 8). According to [36], decreasing *b* should be associated with larger cells that have reduced SA/V ratios, thus increasing the effects of SA limits on metabolic scaling (see Section 2.3.4). However, although above the common midpoint body mass (as specified by a common *L*), this mechanism entails decreased metabolic rates in larger cells, as required by simple geometric cell-size theory (see Section 2.1.1), the opposite occurs below this intersection point, which contradicts this theory and the effect of cell size on *R*/*M* depicted in Figure 7. Unfortunately, this inconsistency has yet to be resolved.

So far in my review, I have emphasized potentially important SA/V effects at the cellular and whole-body levels on metabolic rate and its scaling with body mass, but other mechanisms may also be involved, as discussed next.

## 3. Mechanisms Underlying Cell-Size Effects on Metabolic Rate and Its Scaling with Body Mass

In this section, I discuss various mechanisms by which cell size may affect whole-body metabolic rate and its scaling with body mass. These include effects of cellular SA/V constraints, intracellular resource-transport limits, cell composition, and the resource demand of whole-body growth as mediated by the cellular mode of growth. In effect, I use a cellular perspective to apply all four of the major modal mechanisms (SA: surface area; RT: resource transport; SC: system composition; and RD: resource demand) specified by the “contextual multimodal theory” (CMT) of metabolic scaling [14,18] (see also Section 3.6).

### 3.1. Surface Area/Volume Effects

As briefly described in Section 2.1.1, SA/V effects may operate via limits on exchange of resources and metabolic wastes between the interior of cells and their external environment. As isomorphic cells increase in size, their SA increases allometrically (*b*~2/3) with cell V or *M*, and thus rates of resource uptake, ion exchange, and metabolic waste excretion that affect metabolic rate (*R*) should also scale allometrically (*b*~2/3). Although plausible, the cell SA theory of metabolic scaling requires further testing. Comparative tests of how unicellular metabolic scaling is affected by changes in cell size and shape (and thus SA/V ratios) would be especially useful in this regard. One can make two predictions. First, although allometric metabolic scaling should be exhibited in relatively large cells because of SA limits on supplying resources to large volumes of metabolically active cytoplasm and organelles, these limits should be minimal or nonexistent in very small cells with very high SA/V ratios where cellular demand is amply met by resource supply [169]. Indeed, as expected, very small cells often exhibit steep metabolic scaling possibly related to resource demand rather than supply (*b ≥* 1) [169,170,171]. Second, increased SA/V ratios made possible by increased flattening or elongation of cell shape and/or increased folding of the cell membrane surface should facilitate the support of metabolism by resource uptake and waste removal [53,172]. Shape-shifting-related increases in SA may allow metabolic scaling exponents (*b*) to exceed 2/3 and even approach 1 in unicellular organisms [14,53,164,172]. Increases in the SA of intracellular membranes may also affect cellular *R* and its scaling with cell V or *M* [53,172,173,174,175,176].

Furthermore, one may ask whether cell shape affects *R* and its scaling with *M* in multicellular organisms as well. Given the current lack of relevant data, I can only offer some speculation to stimulate further research. Consider that in most animals, a major part of their body mass constitutes muscle tissue consisting of elongated cell ‘fibers’, which are required for effective muscular contraction and extension, and thus locomotor movement. It is therefore reasonable to suppose that the high SA/V ratios of muscle fibers may contribute to whole-body metabolic scaling exponents (*b*) that exceed 2/3. In support, many studies have shown that as locomotor activity increases, and muscular metabolism becomes an increasingly greater portion of total body metabolism, the scaling of whole-body metabolism also becomes significantly steeper (*b* approaching 1) [9,29,50,163,175,177,178]. As expected, the steepening of metabolic scaling during strenuous exercise is especially enhanced in athletic (muscular) species [14,104,163,177].

In addition, many cells in the extensive structural and vascular tissues of the roots, stems, and leaves of tracheophytes (vascular plants) are elongated, with relatively high SA/V ratios. Therefore, one may ask how much an elongated cell shape contributes to the relatively steep ontogenetic metabolic scaling of many plants (see Figure 1 legend and [105,141,142,143]). During the ontogeny of vascular plants, many cells not only expand in size (see Section 2.3.2) but also become more elongated [179]. According to the cell SA theory of metabolic scaling, increased cell expansion relative to cell multiplication should cause metabolic scaling exponents (*b*) to decrease (see Section 2.3.1, Figure 2 and Figure 7), but this may be mitigated by cell elongation that helps maintain relatively high SA/V ratios. In short, future tests of the cell SA theory of metabolic scaling should consider not only cell size, as emphasized in this review, but also cell shape.

Another explanation for the common observation that large cells exhibit lower *R*/*M* values than do smaller cells is that they require less metabolic energy to maintain ionic gradients across their membranes, because of their low SA relative to cell V [20,23,25,27]. However, is ionic regulation costly enough to make a difference in the *R*/*M* of small versus large cells? In various kinds of mammal tissues, the metabolic costs of ion transport (Na^+^-K^+^-ATPase activity) makes up 1–70% of the total oxygen consumption of their cells [180,181]. Clearly, the energetic cost of cross-membrane ion transport varies greatly among cell types, and thus could greatly affect analyses examining relationships between cell size and *R*/*M*. In addition, the proportional costs of cellular ion transport in other kinds of organisms remains to be determined. Nevertheless, the cost of ion transport relative to cell membrane SA in various crustaceans, fishes, and birds has been shown to be less in larger muscle cells, as predicted [182,183].

### 3.2. Intracellular Resource-Transport Effects

Several investigators have suggested that the ability of cells to meet their metabolic demand by resource supply may become increasingly limited as they increase in size, not only because of SA/V constraints, but also because intracellular resource transport (RT) to all regions of a cell may become more difficult as they become larger [38,184,185]. This explanation represents a cellular version of RT theory proposed to explain hypometric metabolic scaling at the organismal level [14,184,186,187]. This RT explanation is plausible but has yet to be supported with direct evidence. Problematically, the relative importance of SA and RT mechanisms in causing correlations between cell size and *R*/*M* and/or the metabolic scaling exponent (*b*) are difficult to distinguish [18]. Methods should be developed to distinguish the effects of cellular SA and RT mechanisms on metabolic scaling, as has been carried out by analyzing the geometry of body shape in aquatic animals with cutaneous respiration [107,188,189,190,191].

### 3.3. Cell and Body Composition Effects

As suggested in Section 2.3.3, hypometric metabolic scaling arising from ontogenetic increases in the relative proportion of body mass consisting of tissues with relatively low metabolic activity (i.e., system composition (SC) theory; see [14]) may often be largely the result of cell expansion. Furthermore, this SC effect may occur at not only the organismal level but also the cellular level, as in animal adipose tissue and plant structural tissues that accumulate large amounts of metabolically inert materials during cell expansion (see Section 2.3.3). The range of applicability of these cellular and organismal SC effects of cell expansion across taxa and tissue types now needs to be tested.

### 3.4. Effects of Resource Demand by Growth, as Mediated by the Cellular Mode of Growth

As supported by evidence presented in Section 2.3.2, somatic growth rate may relate to cell size and its covariation with body size. Organisms with large cells often grow slower than those with smaller cells [20]. This pattern is supported by negative associations between genome size (a proxy for cell size) and rates of growth and development in a variety of organisms (reviewed in [28,56]). In addition, rapid growth tends to be associated with cell multiplication, whereas slow growth often involves cell expansion (see Section 2.3.2). Since steep ontogenetic metabolic scaling is often associated with rapid postembryonic growth (see Section 2.3.2), it follows logically that cell size and growth during ontogeny should importantly affect metabolic scaling, a mechanism requiring further testing in a variety of organisms.

### 3.5. Cell-Size Variation in Time and Space

So far, most of my discussion regarding mechanisms has assumed homogeneity of cell size and how it changes during development in different tissue types. This seems reasonable as a first approximation because several studies have shown coordinated evolutionary and phenotypically plastic changes in cell size among multiple tissue types in animals [20,40] and plants [192,193], possibly mediated at least in part by genome size [56,192]. Nevertheless, cell-size changes in relation to increasing body size across ontogeny or phylogeny may differ significantly among some tissue types [40,193,194]. Furthermore, different tissue types [60] and developmental stages [39] may show different relationships between *R*/*M* and cell size. Therefore, future research should consider possible heterogeneity of effects among tissue types. A recent theoretical model posits that cell-size heterogeneity affects metabolic scaling [37], but it is limited by only considering fractal variation in cell size, a hypothetical pattern that has yet to be supported with empirical data.

### 3.6. A Holistic Hierarchical View: Linking Mechanisms at the Cell, Organ, and Whole-Organism Levels

**Figure 9:**
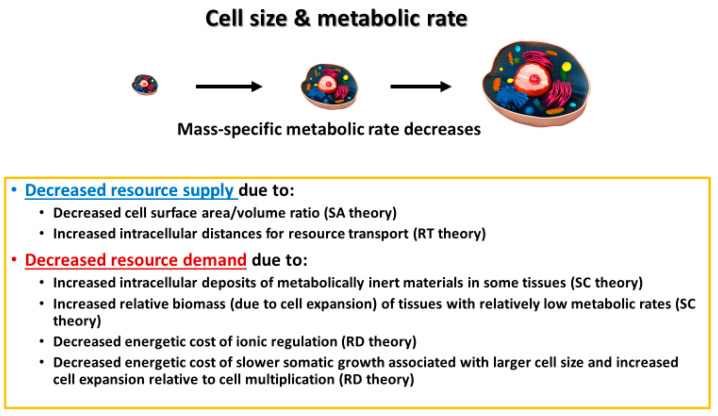
Summary of effects of cell size and expansion on mass-specific organismal metabolic rate, classified according to the major modal mechanisms of the contextual multimodal theory (CMT) of metabolic scaling [14,18], including surface area (SA) theory, resource transport (RT) theory, system composition (SC) theory, and resource demand (RD) theory. SA and RT mechanisms limit resource supply, whereas SC and RD mechanisms relate to resource demand. Cell picture from [198].

A focus on cell size and its relationship to body size presents a promising way of synthesizing the SA, RT, SC, and RD modal mechanisms causing metabolic scaling, especially within species during ontogeny, but perhaps, at least in part, across species as well. Developmental cell expansion may cause decreases in *R*/*M*, and thus hypometric ontogenetic metabolic scaling (*b* < 1) via (1) decreased resource supply to metabolizing cells, as a result of greater SA- and RT-related limits in larger cells; and (2) decreased mass-specific resource demand because of (a) increases in both intracellular deposits of metabolically inert materials and relative masses of tissues with relatively low metabolic activity (SC effects) and (b) an association of larger cells and/or cell expansion with lower relative costs of ionic regulation and slower rates of energetically expensive growth (RD effects) (summarized in Figure 9). In short, a cell-size perspective nicely shows how a comprehensive understanding of metabolic scaling requires an examination of mechanisms related to both resource supply and demand (see also [9,14,20,50,163,195,196,197]).

In addition, a comprehensive understanding of metabolic scaling requires a hierarchical view involving effects not only at the cellular level, as emphasized in this review, but also the intracellular, organ, and whole-organism levels (see also [14,30]). Such a perspective situates cell-size effects on metabolic rate in a holistic context, thereby helping to explain variation in the magnitude of these effects as a result of variation in the countervailing influences of other intrinsic (biological) or extrinsic (environmental) factors. After all, metabolic scaling may be affected by both cellular and whole-body systemic effects [30].

For example, it is a challenge for future research to determine how much metabolic scaling relates to cellular versus organismal SA/V constraints. Consider that although metabolic scaling in largely ectothermic insects appears to follow cell-size theory [31,41,72], metabolic scaling in endothermic birds and mammals, as a whole, does not [18,29]. Systemic thermoregulation (involving the compensation of whole-body SA-related heat dissipation by metabolic heat production) appears to dominate the metabolic scaling of endothermic birds and small mammals, causing the scaling exponent (*b*) to be near 2/3 under thermoneutral conditions [50,163] or near 0.5 under cold stress [166], rather than nearly 1 (due to variation in body size being related much more to cell number than to cell size), as predicted by cell-size theory [18,29]. In particular, the scaling exponent for cell size in mammals is only ~0.03–0.05 [5,145], which predicts a metabolic scaling exponent of ~0.98–0.99 (calculated using Equation (2)), far greater than that actually observed [6,50,163,166]. Furthermore, the allometric body-mass scaling of cellular metabolic rate across species of birds and mammals disappears when cells are cultured in vitro, thus suggesting that systemic effects predominate over cellular effects (reviewed in [30]). However, differences in metabolic scaling among orders of birds and mammals appear to be at least partially related to differences in the scaling of genome size (a proxy for cell size) [27]. A recent theoretical model also shows how one may integrate cell-size effects and systemic thermoregulatory effects related to heat dissipation to understand some of the diversity of metabolic exponents (*b*) that have been observed [38].

Organismal SA effects (and associated developmental shifts in body shape) also appear to be of major importance for understanding ontogenetic metabolic scaling in several species of aquatic skin-breathing animals [107,188,189,190,191]. Other organismal factors that may override cell-size effects on metabolic rate and its scaling with body mass include regulated activity level and strong size-selective mortality. Consider that metabolic rate can change drastically in animals based on their activity level (from torpor to rest to strenuous exercise) without any change in cell size. Regulated changes in activity may also affect the body-mass scaling of metabolic rate, being allometric in resting animals, but approaching isometry in torpid and strenuously exercising animals [50,163]. In addition, the freshwater amphipod *Gammarus minus* shows evolutionary and phenotypically plastic changes in ontogenetic metabolic scaling in response to long-term variation in size-selective predation regimes [75,199] or short-term variation in predator cues [200], without any significant change in the scaling of cell size (as indicated by eye-facet size) with eye size (a proxy for body size) [75].

An important intracellular trait that should be considered in the context of relationships between cell size and metabolic rate is genome size. As a rule, cell size so strongly correlates with genome size [28,56] that genome size is often used as a proxy for cell size (as I have done in this review). Increasing genome size is also frequently associated with slower rates of metabolism, development, and cell division (reviewed in [20,28,56,201]). However, it remains to be determined whether these associations involve direct causation or are indirectly mediated by effects of cell size. The latter interpretation has received some support from studies showing that metabolic rate is more related to cell size than genome size [29,32,56]. An increased understanding of relationships between cell size and metabolic rate may also be gained by exploring how both of these traits relate to nucleus size [71] and the numbers and sizes of ATP-producing mitochondria and other organelles critically involved in biosynthesis (e.g., ribosomes) [30,174]. Growing knowledge of how organelle size and number scale with cell size (see e.g., [101,202,203,204,205,206,207,208]) may provide valuable insight into how metabolic rate scales with body size. Even if metabolic scaling is dominated by systemic effects, they must ultimately be manifested “at the cellular level, for example, including induced variation in the function, structure and intracellular densities of mitochondria” [30] (p. 189). Interestingly, nucleus–cell volume ratios decrease with increasing cell size among many kinds of eukaryotic species, a pattern that may be linked to declines in *R*/*M* (hypometric metabolic scaling, *b* < 1), a hypothesis requiring testing [71]. However, this hypothetical pattern is not observed in prokaryotes, which show decreasing nucleoid–cell volume ratios (like eukaryotes [71]), but increasing *R*/*M* (unlike eukaryotes), with increasing cell size (hypermetric metabolic scaling, *b* > 1 [170,171]).

Clearly, a multi-mechanistic, hierarchical approach is required to understand completely the full diversity of metabolic scaling in the living world. The CMT, which embraces multiple theoretical approaches to metabolic scaling, including dynamic energy budget theory [209,210], offers a potentially useful conceptual framework for achieving a comprehensive synthesis [14,18]. The relative expression of the SA, RT, SC, and RD modal mechanisms may be orchestrated by various types of biological regulation, which is briefly discussed next.

## 4. Effects of Biological Regulation on Metabolic Rate and Its Scaling with Body Mass

Early leaders in the study of metabolic scaling believed that systemic regulatory factors are importantly involved [211,212], but this perspective has been neglected until recently [3,14,18,20,109,196,213]. For example, the neuroendocrine system plays an important role in regulating activity level and body temperature, which can in turn profoundly influence the scaling of metabolic rate (as reviewed in [9,14,29,50,163,166,167,177,178]; see also Section 3.6). Given the various ways that cell size and the relative expansion and multiplication of cells during ontogenetic growth can affect metabolic scaling, as documented or hypothesized in this review, it now seems imperative to explore how biological regulation at the cellular level plays a role in these effects. Knowledge of how cell expansion and multiplication are controlled by various hormones, growth factors, cell signaling systems, and genes (including their controlled expression) in both animals and plants has grown rapidly in recent years (see e.g., [3,129,131,132,214,215,216,217,218,219,220,221]). However, this mechanistic knowledge at the cellular level has yet to be applied to our understanding of ontogenetic metabolic scaling at the organismal level, which I believe is a major frontier awaiting highly rewarding exploration.

## 5. Conclusions

Variation in cell size and number in organisms and how it is achieved by cell expansion and multiplication may help explain much (but certainly not all) variation in metabolic rate and its scaling with body mass (see also Section 3.6). A multi-mechanistic approach is required to understand completely the diversity of metabolic scaling [14,18,20]. Nevertheless, the cell-size perspective deserves more attention than it has been given, because it offers a potentially fruitful way to link regulatory and metabolic machinery at the cellular level to the physiology of metabolic scaling at the organismal level (see also Section 4). Future research on metabolic scaling would benefit from integrating biological regulatory mechanisms operating at the cell, tissue, organ, and organismal levels. I recommend that this hierarchical perspective include multidirectional cause and effect relationships, including upward, downward, and reciprocal causation between metabolism, cell size, nucleus size, genome size, body size, growth rate, and other influential intrinsic (biological) and extrinsic (environmental) factors (see also [3,14,56,71,109,196]).

As a result, we may be able to integrate developmental biology and ontogenetic metabolic scaling in mutually beneficial ways. For example, a holistic conceptual framework embracing both of these fields may shed new light on the old question about why organisms often exhibit strong trade-offs between somatic growth and differentiation [81,100,111,112,218,222,223]. The mechanisms underlying this fundamental developmental trade-off may not only help explain but also be better understood in light of commonly observed ontogenetic shifts in metabolic scaling. Consider that early rapid postembryonic growth depends heavily on cell multiplication, which contributes to steep metabolic scaling, whereas later slower growth and cell differentiation are often associated with cell expansion and relatively shallow metabolic scaling (see also Section 2.3.2, Figure 4). The energetic demand of rapid growth and cell proliferation may not only dictate metabolic rate but also depend strongly on metabolic support and control [3,221] and thus the availability of metabolites [3,218]. Shifts from cell multiplication to cell differentiation are sensitive to nutrient availability (as observed in fission yeast, *Schizosaccharomyces pombe* [218], and fruit fly ovaries [224]). Therefore, the body-mass scaling of metabolic rate may serve as a useful energetic indicator of major transitions during ontogenetic development (see also [9,98,99,104,225,226]). Rapid growth associated with cell multiplication is very expensive energetically, thus requiring large increases in metabolic rate, whereas the relatively slow growth associated with cell differentiation and/or expansion appears to be less costly energetically, thus requiring relatively small increases in metabolic rate (cf. [102,223]).

In addition, a cell-size perspective may help bridge the fields of life-history evolution and ontogenetic metabolic scaling. For example, genome size (a proxy for cell size) often correlates positively with propagule size but negatively with propagule number in a variety of animals and plants [56]. Since cell size also often relates to mass-specific metabolic rate, as shown in this review, further research should explore how variation in cell size may cause covariation between metabolic rate and reproductive strategies in organisms. As another example, senescent tissues are often associated with enlarged cells (e.g., [227,228,229,230,231]), which may help explain why aging often involves a reduction in mass-specific metabolic rate (see e.g., [212,232,233,234,235,236]).

In short, a cellular perspective promises to enlighten our understanding of whole-organism development and life histories in multiple ways, including their metabolic (energetic and biochemical), informational (regulatory), structural (histological, anatomical, and morphological), and functional (physiological and biomechanical) aspects.

## Figures and Tables

**Table 1:** Multiple ways that whole-body metabolic rate and its scaling with body mass may relate to cell size.

Metabolic Effect	Possible Mode of Effect	Sources
Mass-specific or mass-corrected metabolic rate	Cellular SA/V geometry ^1,2^	[22,23,25,26,27,28]
Metabolic level (*L*) of body-mass scaling relationship ^3^	Cellular SA/V geometry ^2^	[29] [present study]
Metabolic scaling slope (*b*)	Cellular mode of growth and/or changes in cell composition	[23,24,27] [present study]
	Total cellular SA of body	[21,36]
	Relative influences of cellular and organismal SA vs. organismal V, as mediated by metabolic level (*L*)	[29,36]

^1^ SA = surface area; V = volume. ^2^ Other possible mechanisms (e.g., cell composition, intracellular resource transport, and heterogeneity in cell size) are discussed in Section 3.2 and Section 3.3. ^3^ *L* = mass-specific metabolic rate at the geometric midpoint of the log body-mass range of a metabolic scaling relationship [29,50,51,52].

## Data Availability

Not applicable.
